# Improving the Efficiency of Semitransparent Perovskite Solar Cell Using Down-Conversion Coating

**DOI:** 10.1021/acsami.4c12551

**Published:** 2024-11-11

**Authors:** Damian Glowienka, Chieh-Ming Tsai, Aoussaj Sbai, Dian Luo, Pei-Huan Lee, Shih-Han Huang, Chia-Feng Li, Hao-Wen Wang, Guey-Sheng Liou, Julien Guthmuller, Wei-Fang Su

**Affiliations:** † Department of Materials Science and Engineering, National Taiwan University, 10617, Taipei, Taiwan; ‡ Faculty of Applied Physics and Mathematics, Gdańsk University of Technology, Narutowicza 11/12, 80-233, Gdańsk, Poland; § Department of Materials Engineering, Ming-Chi University of Technology, 243303, New Taipei City, Taiwan; ∥ Department of Chemistry, National Taiwan University, 10617, Taipei, Taiwan; ⊥ Institute of Polymer Science and Engineering, National Taiwan University, 10617, Taipei, Taiwan

**Keywords:** perovskite solar cells, down-conversion, optimization, power conversion efficiency

## Abstract

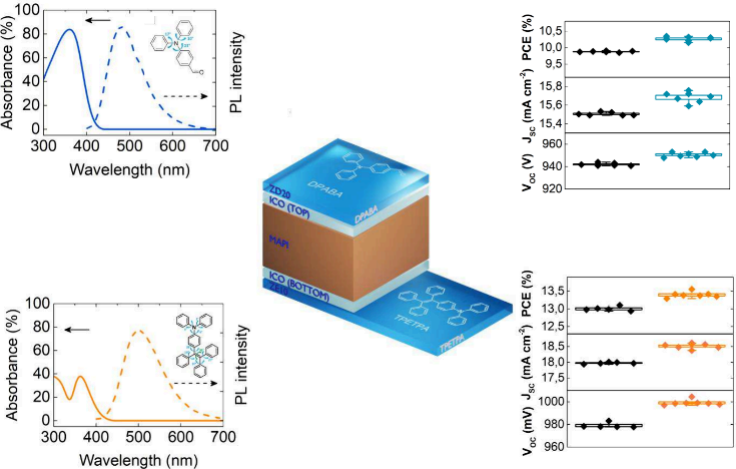

Perovskite solar cells (PSCs) have demonstrated exceptional efficiency, yet surpassing theoretical performance limits requires innovative methodologies. Among these, down-conversion techniques are pivotal in reducing optical losses and enhancing energy conversion efficiency. In this study, optical modeling, including a generalized transfer-matrix optical model, was employed to meticulously assess optical losses in semitransparent PSCs illuminated from the front and rear sides of the device. To reduce these losses, two down-conversion layers, made of *N*,*N*-diphenyl-4-(1,2,2-triphenylethenyl)-benzenamine and 4-(*N*,*N*-diphenylamino)benzaldehyde mixed with polymeric binder, were developed, showcasing initial photoluminescence quantum yields of 60% and 50% as films, respectively. The materials luminescence relies on the effect of aggregation-induced emission, which enhances the fluorescence of the dyes within the binder, providing their films with a unique behavior beneficial for photovoltaic applications. An optimization of these layers was performed, which aimed to reduce UV optical losses by adjusting the film thickness atop the PSCs. The refined down-conversion layers yielded a notable increase in the power conversion efficiency by approximately 0.4% for both the front and rear sides of the PSCs, demonstrating their significant potential in pushing the boundaries of solar cell performance.

## 1 Introduction

Perovskite materials have garnered significant attention in recent years due to their application as absorber materials in solar cells. With ongoing advancements in this field, the current state-of-the-art power conversion efficiency (PCE) of perovskite solar cells (PSCs) has reached a record value exceeding 26%.^1^ However, the achieved performance falls short of the theoretical maximum. According to the Shockley-Queisser model, the optimal PCE for PSCs could reach as high a value as 33.7% for single heterojunctions with a 1.33 eV bandgap. To date, the highest reported PCE utilized a material with a 1.55 eV bandgap,^2^ suggesting a theoretical limit of 31.4% for PSCs under AM1.5G sunlight.

To surpass the theoretical efficiency limits of PSCs, it is crucial to address the issue of unabsorbed light. More than 40% of the incident light energy remains unconverted to electric current, primarily due to parasitic absorption within the solar cell or due to the photon energy being too low to trigger the photoelectric process.^3^ To mitigate these losses and exceed the efficiency threshold, several strategies can be implemented: forming a tandem with another solar cell to broaden the absorption spectrum,^4^ reducing losses associated with the generation of hot-charge carriers,^5^ and enhancing the conversion of unabsorbed photons through spectral modification.^6^ Central to these strategies are the concepts of down-conversion and up-conversion. Down-conversion involves converting photons of high energy that would otherwise be parasitically absorbed into lower-energy photons that are more effectively utilized by the solar cell. Conversely, up-conversion allows the cell to capitalize on photons with energy too low to be absorbed under normal conditions by converting them to photons with a higher and more usable energy. These processes effectively expand the usable spectrum of light, thus improving the overall efficiency of the solar cells.^7^ The optimization of down-conversion materials enables the harvesting of UV light and its conversion into more usable visible light. At sea level, UV light constitutes about 5% of the total solar energy, which also poses a threat to the structural integrity of the perovskite layer, leading to decreased PCE.^8^ Therefore, the down-conversion layer not only boosts the photocurrent by transforming UV light into visible light but also minimizes the amount of harmful high-energy UV light penetrating the organic perovskite layer.^9,10^

Down-conversion materials, including quantum dots, inorganic (rare earth) materials, and organic materials, have shown significant improvements in the PCE of PSCs.^6^ While quantum dots and inorganic materials enhance PCE through superior photoluminescence (PL) capabilities, organic materials, particularly aggregation-induced emission (AIE) molecules, offer a cost-effective alternative with substantial performance benefits due to their high emission characteristics. Up to our knowledge, the highest improvement in efficiency of PSC using organic molecules is already higher than 1% and shows serious improvement of UV illumination stability in the devices.^9^ This makes organic materials promising candidates for enhancing PSC efficiency and stability.^11^ AIE materials display unique behavior wherein their luminescence intensity increases upon aggregation in a condensed state, in contrast to the typical fluorescence of aggregation-caused quenching (ACQ) materials, which diminish when aggregated.^12^ In the aggregated state, molecular vibrations and rotations are restricted, minimizing nonradiative energy loss and, thus, enhancing light emission.^13−15^ Within this framework of enhancing PSC efficiency, two specific AIE molecules, 4-(*N*,*N*-diphenylamino)benzaldehyde (DPABA) and *N*,*N*-diphenyl-4-(1,2,2-triphenylethenyl)-benzenamine (TPETPA), were rigorously tested for their down-conversion capabilities and subsequent application in PSCs. These molecules exemplify a new frontier in photovoltaic materials due to their unique properties, which allow for increased fluorescence in the aggregated state, thus potentially maximizing the spectral usage of solar energy within the cells.

## 2 Experimental and Simulation Section

### 2.1 Materials

Chlorobenzene (CB, >99.0%), *N*,*N*-dimethylformamide (DMF, 99.8%), dimethyl sulfoxide (DMSO, >99.9%), and isopropyl alcohol (IPA, 99.8%) were purchased from Acros Organics. Diethyl ether (99.0%) and ethanol (99.99%) were obtained from Fisher Chemical. Polyethylenimine (PEI, branched, MW 25000) was acquired from Alfa Aesar. Targets with 97 wt % In_2_O_3_ and 3 wt % CeO_2_ (CeO_2_/In_2_O_3_, ICO), methylammonium iodide (MAI), lead iodide (PbI_2_, 99.9985%), and [6,6]-phenyl-C61-butyric acid methyl ester (PC_61_BM, 99.0%) were sourced from FrontMaterials Co. Ltd. 4-(*N*,*N*-Diphenylamino)benzaldehyde (DPABA, 96%, Synthonix), poly(tetrafluoroethylene vinylether) (TFEVE) (Zeffle GK570, Daikin), ethyl acetate (EA, ACS grade, Macron), and tetrahydrofuran (THF, ACS grade, Macron) were also used. *N*,*N*-Diphenyl-4-(1,2,2-triphenylethenyl)-benzenamine (TPETPA) was synthesized by Dr. Guey-Sheng Liou’s lab.^13^

### 2.2 Fabrication of the Semitransparent Perovskite Solar Cell

Commercial indium tin oxide (ITO) glass (electrode) was used as the front transparent electrode, and it was ultrasonically washed using, respectively, acetone, methanol, and isopropanol. Further the glasses were cleaned with O_2_ plasma treatment for 15 min to improve the surface morphology of the ITO substrates.^16^ The NiO_*x*_ nanoparticles used for hole transport layer (HTL) (20 mg mL^–1^ in deionized water) were spin-coated onto cleaned ITO glass at 2500 rpm for 60 s and annealed at 160 °C for 30 min in air. For the synthesis method of NiO_*x*_ nanoparticles, we followed the procedure from our previous work.^17^ P3HT-COOH solution was dissolved in DMF with concentration 1.5 mg mL^–1^, stirred over a week, and then diluted to 0.5 mg mL^–1^ concentration before coating. P3HT-COOH was coated with 4000 rpm, 4000 rpm/s, for 35 s and annealed 150 °C for 20 min to fully remove the solvent. The perovskite precursor (1.2 M) solution was prepared by mixing MAI and PbI_2_ (in a 1:1 molar ratio) in 1 mL solvent mixture of DMF/DMSO (5:2 volumetric ratio). The perovskite precursor was spin-coated onto the HTL at 4500 rpm for 30 s in a nitrogen filled glovebox. At 15 s into the spinning process, 300 μL of diethyl ether was dropped onto the perovskite film by an antisolvent technique. Further, all the samples were sequentially annealed on a hot-plate first at 70 °C for 1 min and second at 100 °C for 2 min. Afterward, the PC_61_BM solution (2.5 wt % in chlorobenzene) was spin-coated onto the dark-brown perovskite layer at 1000 rpm for 30 s to form an electron transport layer (ETL). Finally, the buffer layer (TBAOH-SnO_2_), which followed the procedure from previous work,^18^ was spin-coated at 1500 rpm for 30 s on ETL. For the preparation of semitransparent perovskite solar cell, Ce-doped indium oxide (ICO) electrode was deposited onto ETL to create an active area of 0.09 cm^2^ using DC sputter equipment (Kao Duen Tec. Co.). For the preparation of ICO rear electrode, a target material was used using 97 wt % In_2_O_3_ and 3 wt % CeO_2_. The samples were transferred into the chamber of the sputtering machine, and to eliminate the excess oxygen and moisture, the chamber was evacuated until the pressure dropped below 2 × 10^–6^ Torr. For the process the pure argon and oxygen/argon in volume ratio of 1:99 gas mixture. In order to prevent the destruction of the perovskite layer and PC_61_BM ETL from the environment of high temperature and high-energy particles, the ICO electrode was deposited by DC-sputter technique with the low sputter power of 50 W under working pressure of 3 mTorr. The process atmosphere with different oxygen flow to total flow ratios (*r*(O_2_) = O_2_/(Ar + O_2_)) was applied during the sputtering process to get optimized *r*(O_2_) equal to 0.13%. The flow rate of pure Ar and O_2_/Ar mixture gas was equal to 26 and 4 sccm, respectively.

### 2.3 Fabrication of the Down-Conversion Film

An appropriate amount TFEVE in a glass sample vial was heated in a 90 °C oven for 2 h to remove butyl acetate solvent. Then different amounts of DPABA with 3 mL of EA or TPETPA with 3 mL of THF were added into the vial. With 30 min ultrasound bath treatment, the solution becomes clear light yellow. 200 μL of the solution was spin-coated on quartzes, silicon wafers, or transparent electrode of solar cell devices for each analysis. The spinning conditions were 2000 rpm, 120 s. For long-term durability studies, the rear side of the PSCs requires additional encapsulation with glass coverslips. This encapsulation is essential for the fabrication of down-conversion layer, but overall, the coverslip glass has a minimal impact on the optical calculations due to its large thickness compared to coherent layers.

### 2.4 Characterization Methods

The ellipsometry (SE-950, Radiation Technology Co., LTD) measurements have been done at a constant angle of 69° in order to measure the change in polarization state caused by the sample reflection (psi and delta) in the wavelength function. The PL measurements for films were done using 1 cm^2^ quartz samples and for solution using a quartz tube. With a 355 nm laser source, the PL signals were detected by a PL signal analyzer (C10027, Hamamatsu) with an integrating sphere. The transparency of samples was measured by a UV–vis instrument (V-650, JASCO) on quartz substrates. After taking a blank as background, the transmittance of the samples was measured from 250 to 800 nm. External Quantum Efficiency (EQE) curves of the devices were measured by using an EQE system (LSQE-R, LiveStrong Optoelectronics). The photocurrent–voltage (*J*(*V*)) curves of devices were measured by using a source meter (Keithley 2410) with 100 mW cm^–2^ illumination of AM1.5G solar simulator (YSS-150A, Yamashita Denso).

### 2.5 Generalized Transfer Matrix Model

Generalized matrix method (GTM) is able to calculate the total transmittance, reflectance, internal light energy flux, and internal light energy absorption, including the case of oblique incidence.^19^ Stratified structures with isotropic and homogeneous media and parallel-plane interfaces are described by matrices because the equations governing the propagation of the electric field are linear and the tangential component of the electric field is continuous. Also, all calculations are considered for unpolarized light. More details can be found in the Supporting Information.

### 2.6 TDDFT Computational Model

The quantum chemical calculations were performed with the program Gaussian 16.^20^ Density functional theory (DFT) was employed to calculate the geometry and the harmonic vibrational frequencies of the ground state, while time-dependent DFT (TDDFT) was used to compute the singlet excited-state properties (i.e., energy, transition dipole moment, geometry, and vibrational frequencies). All DFT/TDDFT calculations made use of the def2-TZVP basis set^21,22^ and of the exchange-correlation functional MN15.^23^ Density functional dispersion corrections were included to MN15 using the GD3BJ model^24^ and the parameters reported in Goerigk et al.^25^ The vibrational frequency calculations confirmed that all of the optimized geometries correspond to true minima of the potential energy surfaces. The effects of the solvent (ethyl acetate, ϵ = 5.9867) were taken into account by the polarizable continuum model^26^ (PCM) using the default integral equation formalism of the PCM. The TDDFT excited-state properties in solution were obtained from the conventional linear response (LR) theory, employing the nonequilibrium procedure of solvation for the vertical excitation energies at the ground-state geometry (i.e., corresponding to the process of absorption) and using the equilibrium procedure of solvation for the geometry optimizations and vertical emission energies. In addition to the LR results, the vertical absorption and emission energies in solution, associated with the lowest singlet excited state, were estimated using the state-specific (SS) approach^27,28^ as implemented in Gaussian 16 via the External Iteration keyword. The charge density differences (CDDs), molecular orbitals, and Δr descriptor^29^ were computed and visualized with the Multiwfn program.^30^

## 3 Results and Discussion

### 3.1 Optical Analysis of Semitransparent Perovskite Solar Cell

**Figure 1:**
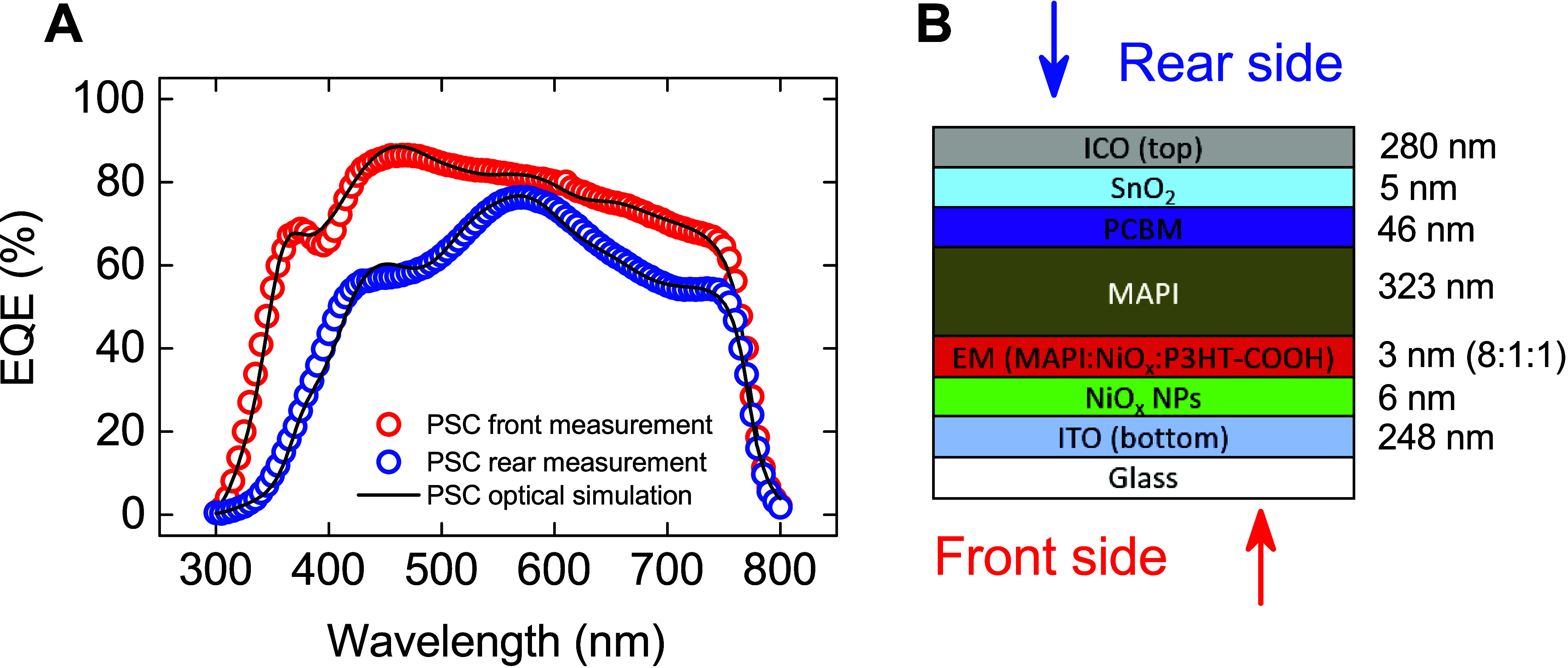
(A) The experimental and simulation results of EQE for the front (red color) and rear (blue color) sides, and (B) the optical structure of PSCs used for both simulations with respected thickness of all layers.

In the fabrication of semitransparent PSCs employing CH_3_NH_3_PbI_3_ based perovskite, a layered architecture consisting of glass/ITO/NiO_*x*_/P_3_HT–COOH/perovskite/PC_61_BM/SnO_2_/ICO was employed as reported in our previous study.^31^ To unravel the intricacies of optical losses within these configurations, a suite of optical modeling techniques was deployed. The characterization of each constituent layer was achieved through ellipsometry and UV–visible spectroscopy, facilitating the determination of their complex refractive indices across a spectrum of wavelengths. Subsequent application of the GTM optical model provided a means to correlate with EQE measurements from both sides of the devices, as illustrated in Figure 1A. This analytical process necessitated refined adjustments to the layer thicknesses, based on profilometry data, to ameliorate inconsistencies in layer uniformity and to enhance measurement accuracy.

In furthering the development of semitransparent PSCs, an effective medium (EM) approach^32^ was employed to integrate a composite optical layer combining the complex refractive indices of perovskite, NiO_*x*_, and P_3_HT–COOH in an 8:1:1 ratio, illustrated in Figure 1B. This configuration resulted in a quasi-inactive (”dead”) perovskite layer characterized by its high absorptivity, which significantly influenced the photocurrent.^33^ The agreement between the experimental and the simulation data validated the chosen modeling parameters. Charge collection efficiency (CCE) was meticulously calculated to delineate the losses of charge carriers across the light spectrum for both the front and rear sides of the device, achieving CCE values of 99.07% and 94.55%, respectively. Throughout these assessments, identical optical configurations were maintained with only the direction of light being modified.

**Figure 2:**
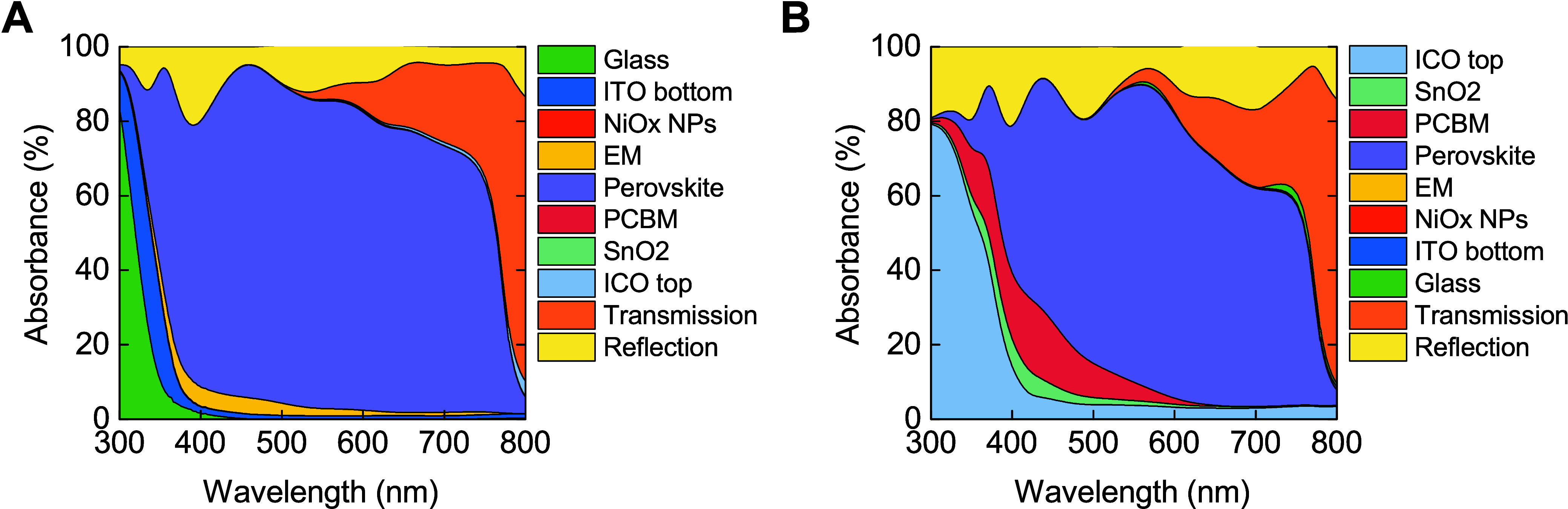
Optical loss analysis for the (A) front and (B) rear side of the semitransparent PSC. The colors are kept the same for all the layers. The order changes depending on the direction of light illumination.

Upon configuring the GTM model, this allows a comprehensive examination of the optical losses in PSCs. Table 1 methodically details the photocurrent absorbed by each constituent layer on both illumination sides. The recorded short-circuit photocurrent density (*J*_sc_), derived from EQE analyses, is equal to 19.38 mA cm^–2^ and 15.35 mA cm^–2^ for the front and rear exposures, respectively. The spectral analysis applies from 300 to 800 nm, consistent with the AM1.5G solar spectrum. Predominant losses are observed in the UV wavelength range on the front side, mostly due to parasitic absorption phenomena associated with the glass and ITO bottom electrodes. As illustrated in Figure 2A, a significant 90% of light within the 300 to 350 nm spectrum is absorbed in this region, which contributes to the overall photocurrent losses exceeding 0.56 mA cm^–2^. Additional losses from the EM layer on the front side result in 0.54 mA cm^–2^ of photocurrent in the visible light region. Efforts to mitigate these losses focus on enhancing the interface between the HTL and the perovskite material, echoing strategies delineated in prior studies.^34^

**Table 1:** Optical losses from the GTM model for the (A) front and (B) rear light illumination side of the semitransparent PSC

Item	Front side losses (mA cm^–2^)	Rear side losses (mA cm^–2^)
Glass	0.11	0.03
ITO (bottom)	0.45	0.05
NiO_*x*_	0.01	0.00
EM (MAPI/NiO_*x*_/P_3_HT–COOH)	0.54	0.03
MAPI	19.53	16.18
PC_61_BM	0.02	1.33
SnO_2_	0.02	0.43
ICO (top)	0.20	1.57
Reflection	2.28	3.53
Transmission	4.15	

For the rear side of the PSCs, significant optical losses predominantly occur within the ICO top layer, which is responsible for over 1.5 mA cm^–2^ of lost photocurrent in the 300 to 400 nm wavelength range, peaking at approximately 80% absorption, as demonstrated in Figure 2B. Further losses are attributed to the layers of PC_61_BM and SnO_2_, contributing to reductions in *J*_sc_ of 1.33 mA cm^–2^ and 0.43 mA cm^–2^, respectively. EQE analysis indicates that almost no photons with wavelengths of up to 400 nm are absorbed by the perovskite layer, a result of the parasitic absorption characteristics of the ICO, PC_61_BM, and SnO_2_ layers. The light that is not absorbed by these layers is either harnessed by the perovskite material, thereby enhancing the photocurrent extracted from the PSCs, or it is lost through transmission and reflection. These transmission and reflection losses are substantial, with the former inducing a loss of 2.28 mA cm^–2^ and the latter 3.53 mA cm^–2^ from the front and rear sides, respectively. The GTM model suggests that this scenario epitomizes the total back reflection across each layer within the stack for each specific wavelength. Moreover, a considerable portion of light is transmitted through the semitransparent structure of the PSC, especially at wavelengths exceeding 600 nm. Knowing the optical losses on both sides of the PSC, we can identify the most suitable optimization techniques. Here, we address the optimization of UV light losses attributed to parasitic absorption on both sides of semitransparent PSCs. This optimization will be realized by implementing down-conversion layers specifically designed to mitigate the UV light losses distinct from each side of illumination in the PSCs under study.

### 3.2 Chemistry of the Down-Conversion Materials

In the development of PSCs, two dye molecules, TPETPA (”E”) and DPABA (”D”), are utilized for their ability to harvest UV light and convert it to visible light. These dyes are integrated with a polymeric binder, TFEVE (”Z”), to stabilize the layer and support the aggregation properties of the AIE molecules. Various concentrations of DPABA and TPETPA are dispersed in the TFEVE binder and solvent for spin-coating to form ZE and ZD down-conversion films. The detailed formulations are listed in Tables S1 to S4 (Supporting Information). The compositions of both ZE and ZD thin layers follow a set mass ratio, which is indicated by the numbers adjacent to the ZE or ZD abbreviations; for example, ZE10 indicates a composition of 10 wt % TPETPA to 90 wt % TFEVE. The chemical structures of down-conversion molecules and binder are illustrated in Figures S2C and S2D (Supporting Information), respectively. Importantly, variations in the ratio of binder to active material do not alter the film thickness after the spin-coating, as shown in Tables S1 and S3 (Supporting Information). Additionally, all of the materials are spin-coated directly onto either the front or rear sides of the device.

We need to disperse the down-conversion molecule homogeneously in a binder solution to make a film from it. Here we explain the selection of different binders for the DPABA dispersion, such as polystyrene (PS) and TFEVE as examples. Figure S1(A) (Supporting Information) presents the absorption spectra of DPABA in both PS and TFEVE matrices. A distinct hypochromic shift is observed, signifying a transition from the 380 nm peak absorption to approximately 360 nm, which is attributed to the solvation of DPABA transitioning from its solid state. This shift is primarily due to the weakening of intermolecular interactions, leading to intramolecular charge transfer (ICT) within the π-orbital of the molecule. Further elucidation of these phenomena is depicted in Figure S1(B) (Supporting Information), which delineates the photoluminescence spectrum of DPABA in similar environments. The emission undergoes a bathochromic shift prompted by the OH functional group in TFEVE exerting a significant influence on the central nitrogen atom, altering the angular disposition of the benzene carbon-N-benzene carbon linkage. This alteration substantially modifies the aggregation condition of DPABA, resulting in a bathochromic shift coupled with a broadened emission profile. This broadening and shift in the emission spectrum illustrate the complex interaction between molecular structure and environmental factors within the photophysical behavior of DPABA. Thus, for the purpose of large quantum yield, we chose TFEVE as a binder for the down-conversion film.

The optical properties of these down-conversion films have been extensively characterized through UV–vis and PL emission spectroscopy, as depicted in Figure S2 (Supporting Information). Notably, higher concentrations of the TPETPA molecules result in increased absorbance within the 300 to 400 nm wavelength range for the ZE film series, with the highest absorption peaks occurring at approximately 310 and 370 nm. The peak of PL emission typically manifests at around 500 nm and exhibits minimal variance in both wavelength and peak intensity across different TPETPA concentrations, indicating stable aggregation properties of TPETPA within the TFEVE binder despite concentration changes. Conversely, the ZD film series exhibits a linear relationship between DPABA concentration and absorption, where increased concentrations lead to a plateau in the absorption peak, reflecting the sensitivity of DPABA’s aggregation condition to its concentration in TFEVE. Notably, the PL behavior of ZD films is typical, showing maximal emission at approximately 480 nm for ZD40. Furthermore, the PLQY of these materials shows a declining trend as the concentration increases, suggesting saturation effects despite the increase in the absorption.

We did quantum chemical calculations for DPABA and TPETPA molecules to get insight into their light absorption and emission phenomena. These analyses aimed to elucidate the nature of the electronic transitions that govern the absorption and emission properties of these films. The computational studies were executed on isolated molecules, both in a vacuum and in the solvent ethyl acetate, employing the polarizable continuum model (PCM). This methodology accounts for polarization effects derived from the molecule environment, although it does not consider specific molecular interactions, such as hydrogen bonding, that may occur within the film or solution. This approach facilitates further understanding of the fundamental electronic properties influencing the optical behavior of these two dye molecules.

Table 2 provides a comparative analysis between the experimental absorption and emission band maxima and the calculated vertical energies of the first singlet excited state S_1_ for DPABA. Notably, solvatochromic shifts (differences in energy between environments in ethyl acetate and vacuum) of −0.31 eV and −0.51 eV are predicted for absorption and emission energies, respectively. This underscores the significant impact of the dye molecule environment on the spectral properties. The energies computed in ethyl acetate align closely with experimental values in both ethyl acetate and the solid phase, exhibiting minimal deviations of approximately 0.1 eV. These results validate the efficacy of the PCM in capturing the interaction between DPABA and its environment in ethyl acetate and approximating these interactions within the film. Furthermore, the Stokes shift for DPABA, critical to the down-conversion process, is precisely predicted by TDDFT coupled with PCM calculations, with a deviation of less than 0.1 eV. These calculations employed the state-specific (SS) approach of the PCM, contrasting with the less accurate results from the standard linear response (LR) method, which suggests a Stokes shift of −0.60 eV as shown in Table S5 (Supporting Information).

**Table 2:** Calculated state-specific vertical absorption energies, vertical emission energies, and Stokes shifts. Experimental absorption and emission wavelength maxima

Item	λ_**max,abs**_ (nm)	λ_**max,em**_ (nm)	**Stokes shift** (eV)
**DPABA**			
Theo. (vacuum)	332 (3.73)	422 (2.93)	–0.80
Theo. (ethyl acetate)	362 (3.42)	512 (2.42)	–1.00
Exp. (film)	355 (3.49)	482 (2.57)	–0.92
Exp. (ethyl acetate)	353 (3.51)	492 (2.52)	–0.99
**TPETPA**			
Theo. (vacuum)	362 (3.42)	646 (1.92)	–1.50
Theo. (ethyl acetate)	377 (3.29)	689 (1.80)	–1.49
Exp. (film)	361 (3.43)	500 (2.48)	–0.95

**Figure 3:**
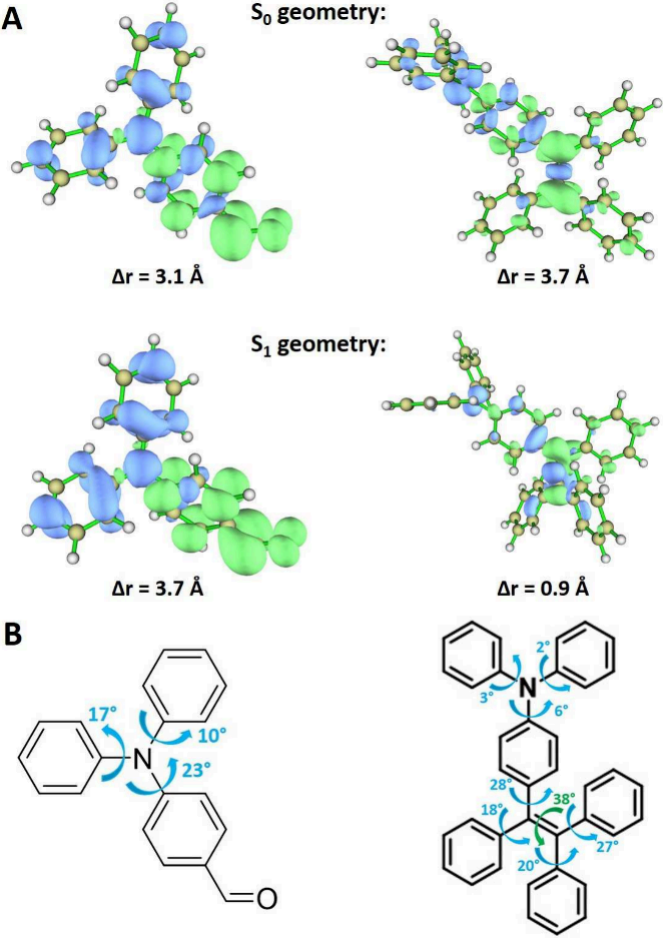
(A) Isosurface of charge density difference and charge transfer index (Δ*r*) for the S_1_ excitation in DPABA (left) and TPETPA (right). Positive (electron) and negative (hole) values are indicated in green and blue colors, respectively. (B) Rotation angles of the main structural differences between the S_0_ and S_1_ geometries of DPABA (left) and TPETPA (right).

Charge density differences (CDDs) illustrated in Figure 3 reveal that the S_1_ state involves a charge transfer (CT) from the nitrogen center and the unsubstituted phenyl groups toward the phenyl-CHO group. This CT character intensifies at the S_1_ state geometry, as indicated by the Δ*r* index values (3.1 and 3.7 Å at the S_0_ and S_1_ geometries, respectively), which estimate the spatial separation between the hole and the electron during the S_0_ → S_1_ transition. Additionally, the calculated geometries indicate that relaxation in the S_1_ state induces a rotation of the phenyl groups ranging between 10° and 23°, with phenyl-CHO exhibiting the most substantial rotation.

The absorption results for TPETPA display a notable resemblance to those of DPABA, characterized by a solvatochromic shift of −0.13 eV and a deviation of −0.14 eV compared to the experimental data obtained in the film. However, the emission properties of TPETPA exhibit a considerably smaller solvatochromic shift of −0.12 eV, starkly contrasting with the −0.51 eV observed for DPABA. Furthermore, the theoretical predictions significantly underestimate the emission energy by 0.68 eV, resulting in an erroneously high Stokes shift of −1.49 eV, as opposed to the −0.95 eV observed experimentally in the film. Charge density differences (CDDs) illustrated in Figure 3, along with the Δ*r* index at 3.7 Å, indicate that the absorbing S_1_ state predominantly features charge transfer (CT) from triphenylamine (TPA) to triphenylethylene (TPE). However, following adiabatic relaxation to the S_1_ minimum, the state’s character alters substantially, predominantly reflecting a local excitation on TPE, as evidenced by the CDD and a Δ*r* index of 0.9 Å. This transformation and the associated energetic stabilization are accompanied by significant rotations within the TPE group, ranging from 18° to 38°, as shown in Figure 3. This discrepancy between calculated and measured emission energies likely stems from the restriction of phenyl rotations within the film, impeding geometrical relaxation in the S_1_ state and, consequently, resulting in elevated emission energies in the solid phase. Since the polarizable continuum model (PCM) does not account for specific interactions between the dye molecule and its environment, the calculations in ethyl acetate yield lower energies. Moreover, the nonemissive nature of TPETPA in ethyl acetate, indicating rapid nonradiative S_1_ → S_0_ decay, aligns with the low S_1_ energy calculated in solution, corroborating the energy gap law. This interpretation is further supported by recent literature on AIE compounds.^13^

**Figure 4:**
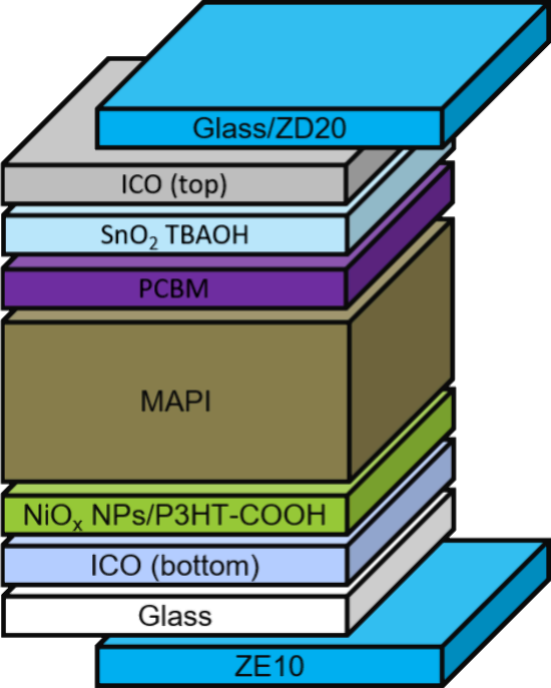
PSCs structure together with down-conversion layers on the front (ZE10) and on the rear (ZD20) side.

For the sake of simplicity, a selection was made to utilize only one of each kind of down-converting film in the development of semitransparent PSCs. Given the specific applications on both the front and rear sides of the samples, the most suitable layers were chosen based on their capacity to optimize the absorption characteristics of PSCs, thereby minimizing optical losses and enhancing photocurrent gain. The evaluations revealed that ZD20 is the most effective for the rear side due to its superior absorption-emission ratio, while ZE10 is similarly advantageous for the front side, as shown in Figure 4. It is critical to acknowledge that the ZD material generally exhibits a higher absorptivity factor, enabling it to capture more UV photons than does the ZE material. This higher quantum yield of PL of approximately 10% for the ZD material underlines its efficacy. However, the lower absorption range of ZE renders it more suitable for the front side of the semitransparent PSCs, leveraging its specific spectral characteristics for optimal application. Consequently, all subsequent optimizations were conducted using ZE10 and ZD20 specifically tailored for application on the front and rear sides of the semitransparent PSCs, respectively.

**Figure 5:**
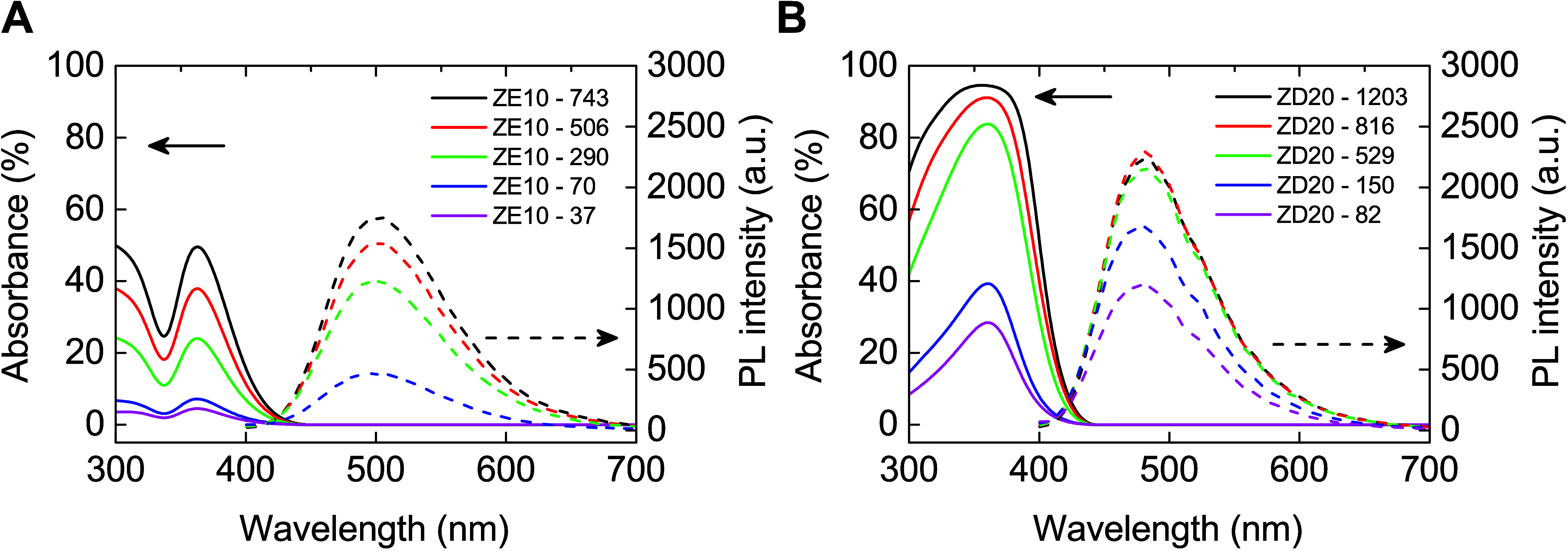
PL and absorption of (A) ZE10 and (B) ZD20 material in different film thickness.

The relationship between film thickness and solution concentration is delineated as approximately linear for both materials, as illustrated in Figure S3 (Supporting Information). Specifically, the thickness of the ZE10 film escalates from 36.8 to around 742.5 nm as the solid concentration varies from 12 to 124 mg mL^–1^. In contrast, the ZD20 film exhibits a thickness increase from 82.1 to 1202.6 nm across the same concentration spectrum. The precise modulation of the down-converting layer’s thickness is crucial for the optimal performance of PSCs, effectively balancing the loss of light that would otherwise be absorbed by the solar cell’s absorber with the benefits of enhanced light illumination provided by the down-converting layer. Consequently, the thickness of these films is employed as a defining parameter for the naming of down-conversion layers; for instance, a ZE10 layer with a 37 nm thickness is referred to as ZE10–37. Figure 5 delineates the variability of the down-converting layers as a function of material concentration in the solvent. It is evident that the absorption increases linearly with the concentration of the mixtures. This is also apparent in the PL spectra, which vary across different film thicknesses. Figure 5A illustrates significant changes in the PL results for varying ZE10 thickness, particularly noticeable at lower film thickness where an increase from 50 to 300 nm leads to a dramatic surge in PL from nearly zero to more than half of its maximum. Further increments in film thickness result in a gradual rise in PL. Table S2 (Supporting Information) validates these subtle enhancements in PLQY, with values ascending from 44% to 51% as the film thickness increases from 37 nm (ZE10–37) to 742.5 nm (ZE10–743), indicating that TPETPA aggregates under different conditions during spin-coating with different TPETPA concentrations in THF solutions, which is a process issue. A consistent trend is noted in the ZD20 layer, where full saturation is reached at a film thickness of 529.3 nm (ZD20–529), as depicted in Figure 5B. However, changes in PLQY are marginal, with only a slight increase from 60% to 61% as the film thickness expands from 82.1 nm (ZD20–82) to 1202.6 nm (ZD20–1203), according to data shown in Table S4 (Supporting Information). This stability in PLQY across varying thicknesses suggests that the aggregation condition of the ZD films remains unchanged throughout the process.

### 3.3 Semitransparent Perovskite Solar Cell with the Application of the Down-Conversion Film

To minimize trial-and-error in determining the optimal thickness of down-conversion materials for use in semitransparent PSCs, the GTM tool is again employed in the previously constructed optical model. The first step involves precisely defining the optical complex refractive index as a function of the analyzed wavelength. This approach mirrors the procedures previously applied to other PSC layers, with specific measurements taken for the ZE10 and ZD20 films. Ellipsometry measurements for both films are detailed in Figures S4A and S4B (Supporting Information). Utilizing Regress Pro freeware,^35^ the change in polarization state (Ψ and Δ) is modeled using the Tauc-Lorentz model,^36^ which aligns well with the experimental data, facilitating the acquisition of complex refractive indices for ZE10 and ZD20 across various wavelengths as shown in Figures S4C and S4D (Supporting Information). Furthermore, absorption measurements of the down-conversion films on quartz substrates are integrated into the GTM model using previously determined *n* and *k* values, as depicted in Figures S4E and S4F (Supporting Information) for ZE10 and ZD20, respectively. The excellent correlation between the GTM optical model and the experimental data substantiates the reliability of the optical parameters. Consequently, these refined models will be utilized to enhance the performance of down-conversion films in the application of semitransparent PSCs on both the front and rear sides. The absorption losses in PSCs, coupled with the effects of the down-conversion material applied to either side, can be quantitatively assessed using the GTM model. This model conceptualizes a system where a coherent down-conversion layer is sandwiched atop an incoherent glass layer and beneath a coherent set of solar cell layers. However, to effectively gauge the potential enhancements afforded by the down-conversion material, its PL emission triggered by UV absorption and subsequent visible light emission must be accurately calculated. In this context, the down-conversion material itself acts as the light source, contributing to the overall gain of the PSC upon application of the film. PL measurements of the down-conversion material were thus interpolated over a broad range of total film thickness, ranging from 50 to 743 nm for ZE10 and from 100 to 1200 nm for ZD20, see Figure S5 (Supporting Information). These findings have been further extrapolated to calculate the power density of the emission spectra, which are then utilized in GTM modeling to compute the potential gains and losses in *J*_sc_ from the PSCs when the materials are applied on either the front (ZE film) or rear (ZD film) illumination sides. This comprehensive modeling approach enables a nuanced understanding of the photovoltaic enhancements achievable through strategic material applications within PSC architectures.

**Figure 6:**
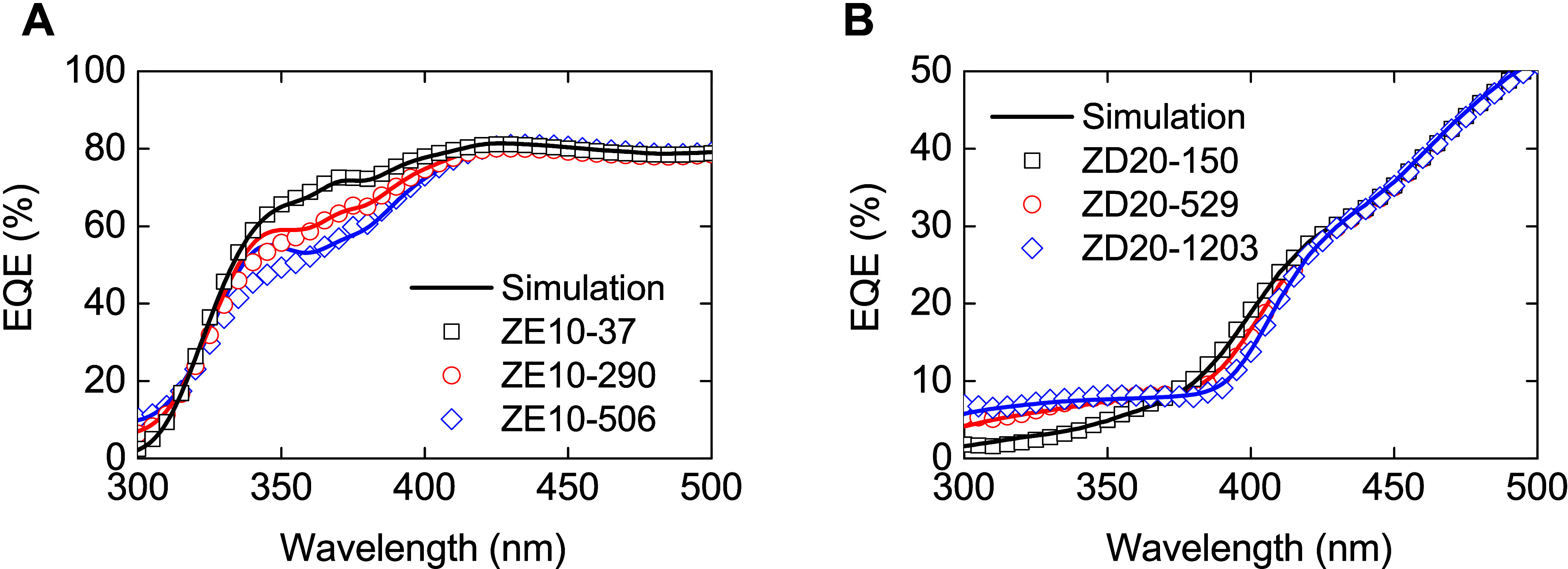
Plots of the changes of EQE of PSCs varied with the film thickness of different materials, (A) ZE10 (front side) and (B) ZD20 (rear side) layers on PSCs.

Another critical factor that necessitates definition within the optical simulation is the real PLQY from the film. In practical terms, PL is gauged using an integration sphere, capturing all photons emitted by the down-conversion film. Within the experimental setup, the down-conversion layer is applied atop the semitransparent PSC, consequently directing only a portion of the emitted light toward the perovskite material. The remainder of the light may either escape laterally from the edges or be back-reflected.^37^ To comprehensively account for these losses, the EQE of PSCs incorporating various thicknesses of the ZE10 and ZD20 layer on the solar cell was measured, as depicted in Figure 6. Notably, for measurements taken from the front side employing ZE10, the absorption of the down-conversion film on PSCs within the 350 to 400 nm wavelength range is clearly evident (see Figure 6A). In the UV region, a modest increase in EQE, approximately 10%, is observed due to the visible light emission from the ZE10–506 film. Conversely, the ZD20 layer, applied to the rear side of the encapsulated semitransparent PSC, enhances the UV light range, achieving up to an 8% increase in EQE across the 300–350 nm wavelength range, as illustrated in Figure 6B. Figure S6 (Supporting Information) shows the full range of both side PSCs measurements with and without ZD20 layer. The disparities in film with 529 and 1203 nm thickness predominantly influence visible light losses due to increased parasitic absorption by the down-conversion film. These observations align with PL measurements across varying thicknesses, which indicate PL saturation in thicker ZD20 films.

Leveraging the equation developed by Rothemund et al.,^37^ which describes the EQE of the solar cell with adjustments for PL and transmission due to the presence of the down-conversion film, the real quantum yield of the down-conversion material (η_DC_) was successfully determined, as detailed in eq 1. This analytical approach enables the precise quantification of the optical enhancements achievable through strategic material applications within PSC architectures.
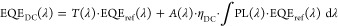
1

In this model, all variables are functions of the wavelength (λ), where ”*A*” represents the absorption and ”*T*” denotes transmission properties. ”PL” stands for photoluminescence emitted by the down-conversion single layer. The EQE parameters are defined for the reference sample without (EQE_ref_) and with (EQE_DC_) the down-conversion layer. This differentiation has been calculated across three distinct thicknesses for ZE10 and ZD20, which are coated on the front and rear sides of the semitransparent PSC, as depicted in Figures 6A and 6B, respectively.

The model fitting aligns exceptionally well with the experimental data, revealing that the quantum efficiency is approximately 30% for ZE10 and 16% for ZD20 within the down-conversion layers on PSCs. Notably, these values show a minimal variation of around 1% across varying thicknesses, mirroring trends previously observed in PLQY measurements for similar film thickness ranges. Moreover, a comparison of the PLQY and quantum yield from EQE fitting indicates a decrease from about 50% to 30% for the ZE10 layer and from 60% to 16% for the ZD20 layer on PSCs. To enhance our understanding of photoluminescence losses in the ZD20 film, we conducted measurements of the film’s PL when applied at different detection angle to analyze all edges of the layer, as detailed in Figure S7 (Supporting Information). It is observed that the emission at 0° from the edge accounts for approximately 1/2 to 1/3 of the total emitted light. This observation aligns closely with the values obtained from the EQE fitting, underscoring a consistent pattern. From these data, it is inferred that only about one-fourth of the light emitted from the down-conversion layer is effectively harnessed by the solar cell, while the remainder is lost at the edges of the coating. This finding is crucial for optimizing the design and application of down-conversion layers in semitransparent PSCs to enhance their efficiency.

**Figure 7:**
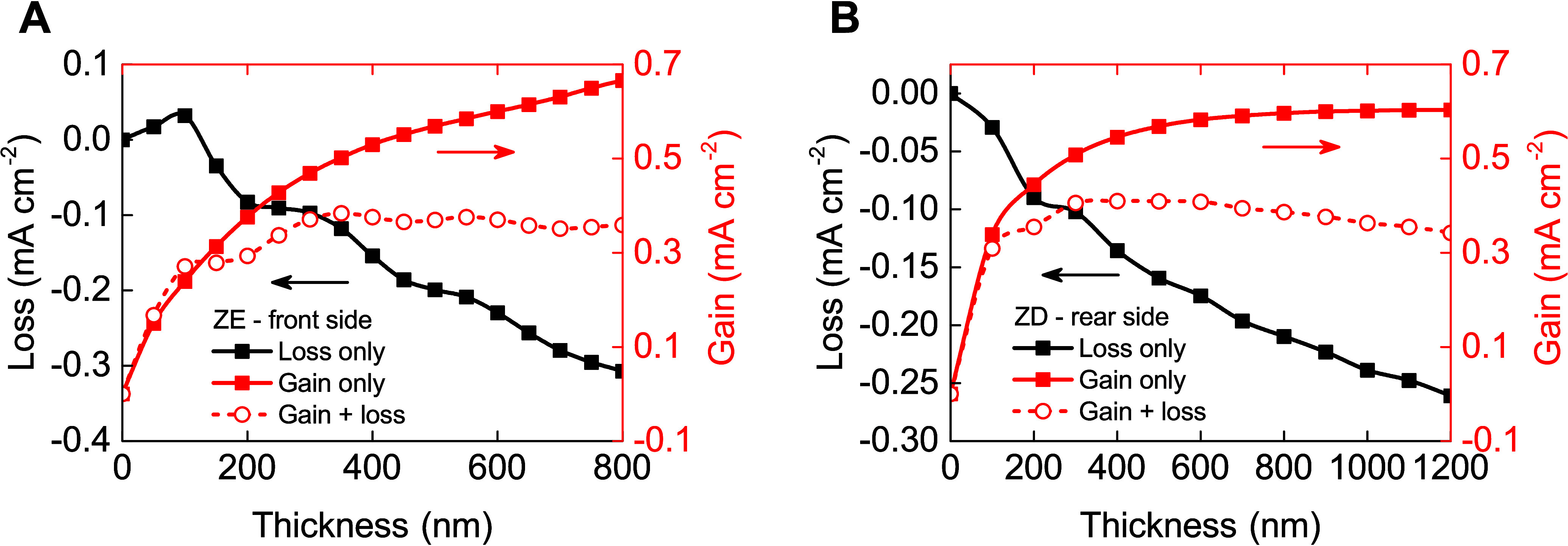
Optical calculation results for the gain from illuminating (A) the front side (ZE10 film) and (B) the rear side (ZD20 film) using different thickness of the down-conversion films.

With precise measurements of light absorption and emission from the down-conversion layer, it is feasible to determine the optimal film thickness for each side of the semitransparent PSC. Figure 7 presents the results from the GTM optical calculations, detailing both the gains and losses in photocurrent when the ZE10 and ZD20 layers are applied to the front and rear sides of the PSC, respectively. Loss calculations are conducted directly using AM1.5G spectra, accounting for parasitic absorption and reflection losses associated with the application of the down-conversion film. Conversely, gains are derived from the interpolated PL emissions from the down-conversion layer directly impacting the PSC. Notably, optical losses increase significantly for thicker down-conversion films. However, for ZE10 layer on solar cell, a slight increase in performance is observed when a 100 nm thickness is applied, enhancing *J*_sc_ by approximately 0.05 mA cm^–2^ (see Figure 7A) due to minor antireflection properties of the coating.^38^ The total gain from applying a 100 nm ZE10 film on the front side is approximately 0.2 mA cm^–2^. The optimal thickness for this film is found to be around 350 nm, yielding an improvement in the *J*_sc_ by 0.38 mA cm^–2^. Figure 7B illustrates a similar pattern for the ZD20 down-conversion film applied to the rear side of the semitransparent PSC. The potential gain could reach as high as 0.6 mA cm^–2^; however, due to losses, the maximum achievable value is approximately 0.4 mA cm^–2^. Therefore, the optimal film thickness for ZD20 on PSCs is determined to be 400 nm. Intriguingly, once the optimum thickness is achieved for either one of the down-conversion layers, the total gain alters negligibly, indicating a plateau in performance enhancements beyond this point.

**Figure 8:**
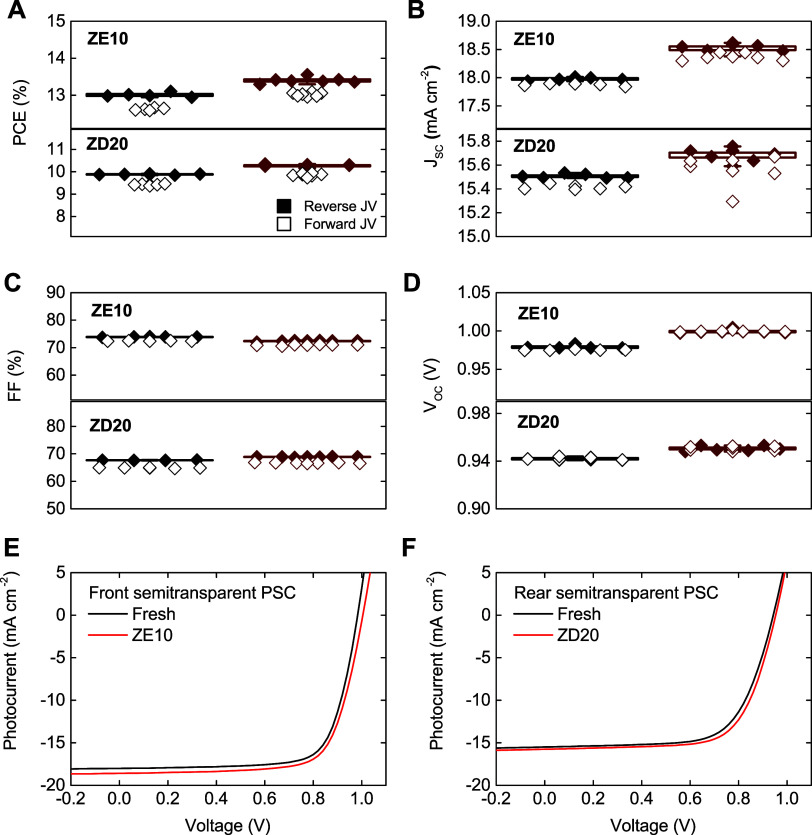
Photovoltaic parameters (A) PCE, (B) *J*_sc_, (C) FF, and (D) *V*_oc_ measured for the semitransparent PSC, measured without additional layers (indicated by black symbols) and with ZE10 and ZD20 down-conversion coatings (indicated by red symbols). Current–Voltage (*J*-*V*) characteristics of champion PSCs are also shown without down-conversion material (in black) and with (E) ZE10 and (F) ZD20 layers (in red).

To ascertain the benefits of optimal film thickness, samples were prepared using the determined optimal thicknesses for the ZE10 and ZD20 layers on PSCs, applied, respectively, to the front and rear sides of the semitransparent PSC. Rather than evaluating multiple devices with varying efficiencies—which could complicate the analysis—a single cell was repeatedly measured by alternately applying and chemically removing the down-conversion layer. The total gain in PCE using ZE10 increased from 13.01% ± 0.04% to 13.40% ± 0.05% for the reverse scan, indicating a net efficiency gain of approximately 0.4% as depicted in Figure 8A. This substantial enhancement by employing AIE material on the semitransparent PSC aligns with gains reported for other nonlanthanide down-conversion materials.^6^

For the ZD20 film applied to the rear side of PSCs, the PCE improved from 9.88% ± 0.02% to 10.27% ± 0.05%, translating to a gain of 0.39% in PCE. This improvement was primarily due to increases in the *J*_sc_, which rose by 0.54 ± 0.04 mA cm^–2^ and 0.18 ± 0.03 mA cm^–2^ for the front and rear sides, respectively, as showcased in Figure 8B. The fill factor (FF) changes were minimal, within the measurement error margin, and are detailed in Figure 8C. However, significant enhancements were also noted in the *V*_oc_, with increases from 979 ± 2 mV to 999 ± 2 mV for the ZE10 on the front side, and from 942 ± 1 mV to 951 ± 2 mV for the ZD20 on the rear side, yielding gains of approximately 20 mV and 9 mV, respectively. These improvements in *V*_oc_ correlate directly with the enhancements in *J*_sc_ attributed to the down-conversion layer, see Figure 8D. Further *J*(*V*) documented in Figures 8E to 8F for the champion devices employing ZE10 and ZD20 layers. Considering the optical characteristics and potential enhancements, it is noted that the maximum possible improvement in *J*_sc_ due to mitigating parasitic absorption is approximately 1.1 mA cm^–2^. On the rear side, losses can exceed 3.3 mA cm^–2^ due to greater absorption in the visible range. Consequently, the realized improvements in *J*_sc_ for the champion devices on the front and rear sides are 0.57 mA cm^–2^ (see Figure 8A) and 0.26 mA cm^–2^ (see Figure 8B), respectively. These values represent 52% and 8% of the total potential gain for the respective front and rear illumination sides.

## 4 Conclusion

In summary, a thorough theoretical analysis was performed to outline all optical losses associated with semitransparent PSCs when illuminated from both the front and rear sides of the device. The most significant losses were identified as parasitic absorption of UV light by the glass and ITO bottom layer, resulting in losses of 0.56 mA cm^–2^ from the front side and approximately 1.5 mA cm^–2^ by the ICO, PC_61_BM, and SnO_2_ layers from the rear side. To address these losses, two down-conversion materials were developed by combining either TPETPA or DPABA of the AIE dye molecule with the polymeric binder of TFEVE. The thin films of these materials exhibited promising initial photoluminescence quantum yields of approximately 50% for the ZE material containing TPETPA and around 60% for the ZD material containing DPABA (ZD). However, direct measurements on the PSC revealed reduced actual quantum yields of 30% for the ZE layer and 16% for the ZD layer. Additionally, TDDFT modeling was employed to elucidate the electronic transitions responsible for the films’ absorption and emission characteristics. The TDDFT calculations effectively estimated the vertical transitions between the singlet excited state S_0_ ⇔ S_1_ and the associated Stokes shifts, revealing that the molecular environment significantly influences the spectral properties of the down-conversion films. Moreover, optical simulations were instrumental in determining the optimal thickness for these films, pinpointing 350 nm for ZE and 400 nm for ZD as being the most effective. Application of these down-conversion layers enhanced the PCE of the PSC by approximately 0.4% for both the front and rear sides separately. Specifically, improvements of 0.54 mA cm^–2^ and 0.18 mA cm^–2^ in the *J*_sc_ were recorded for the front and rear sides, respectively. Furthermore, the *V*_oc_ was incrementally enhanced by 20 and 9 mV, respectively. The materials demonstrate significant potential for application in PSCs due to the visible gain in PCE and the simplicity of the solution coating process, enhancing their appeal for practical deployment. Moreover, improving the techniques for studying and optimizing down-conversion materials is crucial, paving the way to surpass the theoretical efficiency limits.

## Supporting Information Available

Detail description of the Generalized Transfer Matrix simulation tool, additional figures and data for the manuscript (PDF)
