# Supplementary material for: Improving the Efficiency of Semitransparent Perovskite Solar Cell Using Down-Conversion Coating
Source: ACS Appl Mater Interfaces. 2024 Nov 11;16(46):63528–39. doi: 10.1021/acsami.4c12551 (PMC11583132; doi:10.1021/acsami.4c12551)
Supplement: Supplementary file 1 — am4c12551_si_001.pdf [file am4c12551_si_001.pdf]

Supplementary Information

Improving the efficiency of semitransparent perovskite solar cell using down-conversion coating

Damian Glowienka[1,2,#], Chieh-Ming Tsai[1,#], Aoussaj Sbai[2], Dian Luo[3], Pei-Huan Lee[1], Shih–Han Huang[1,3], Chia-Feng Li[1], Hao-Wen Wang[4], Guey-Sheng Liou[5], Julien Guthmuller[2], Wei-Fang Su[1,3,*]

[1] Department of Materials Science and Engineering, National Taiwan University, 10617, Taipei, Taiwan

[2] Faculty of Applied Physics and Mathematics, Gdańsk University of Technology, Narutowicza 11/12, 80-233, Gdańsk, Poland

[3] Department of Materials Engineering, Ming-Chi University of Technology, 243303, New Taipei City, Taiwan

[4] Department of Chemistry, National Taiwan University, 10617, Taipei, Taiwan

[5] Institute of Polymer Science and Engineering, National Taiwan University, 10617, Taipei, Taiwan

[#] equal contribution of the authors

[*] suwf@ntu.edu.tw

I Simulation section - Generalized Transfer Matrix model

The Generalized Matrix Method (GTM) efficiently computes total transmittance, reflectance, and the internal light energy flux and absorption, even under oblique incidence for both coherent and incoherent layers. This method leverages the electromagnetic theory of light to ascertain the reflection and transmission coefficients of electromagnetic fields across multilayer structures through the use of matrix methods. Stratified media, consisting of isotropic and homogeneous materials with parallel-plane interfaces, are aptly described using matrices, due to the linear nature of the electric field propagation equations and the continuity of the electric field's tangential component. This approach is graphically represented, distinguishing between incoherent (red color) and coherent (black color) layers, where a plane wave impacts the multilayer structure from the left, traversing through layers situated between a semi-infinite transparent ambient and a semi-infinite substrate, featuring forward and backward-propagating electric field components.

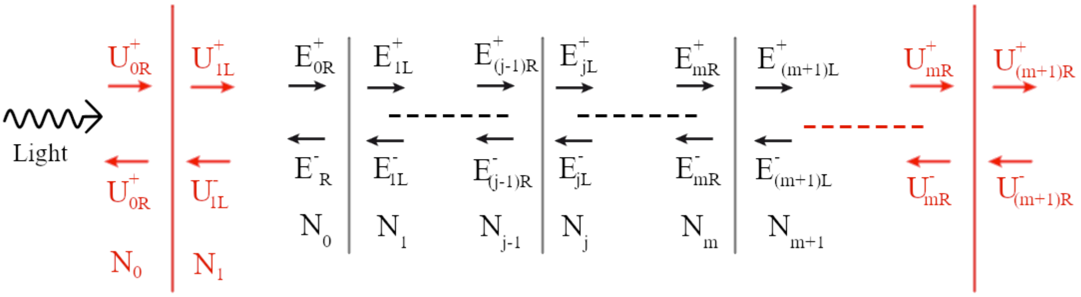

Initially focusing on coherent layers, each layer, labeled j (ranging from *0* to *m+1*), possesses a specified thickness *d* and is characterized by a complex index of refraction or complex dielectric function, which varies according to the wavelength of the incident light. Within any given layer, the optical electric field can be decomposed into two directional components: one propagating in the positive z-direction and the other in the negative z-direction. At any point z within layer j, these components are represented as $E_j^+(z)$ and $E_j^-(z)$, respectively, contributing to the resultant total electric field within that layer.

By using the interface matrix and the layer matrix the total system transfer matrix (scattering matrix) S, which relates the electric field at ambient side and substrate side by

$$\begin{bmatrix} E_{0R}^{+} \\ E_{0R}^{-} \end{bmatrix} = S \begin{bmatrix} E_{(m+1)L}^{+} \\ E_{(m+1)L}^{-} \end{bmatrix}.$$

Where the electric field at each position (z) is a superposition between

$$E(z) = E_j^{+}(z) + E_j^{-}(z)$$

The scattering matrix can be calculated using

$$S = \begin{bmatrix} S_{11} & S_{12} \\ S_{21} & S_{22} \end{bmatrix} = \left(\prod_{v=1}^{m} I_{(v-1)v} L_v \right) \cdot I_{m(m+1)}$$

An interface matrix (matrix of refraction) then describes each interface in the structure

$$I_{i,j} = \frac{1}{t_{ij}} \begin{bmatrix} 1 & r_{ij} \\ r_{ij} & 1 \end{bmatrix}$$

where r_{jk} and t_{jk} are the Fresnel complex reflection and transmission coefficients at the jk interface. All calculations are considered for unpolarized light with the electric field perpendicular to the plane of incidence. The Fresnel complex reflection and transmission coefficients are defined by

$$r_{ij} = \frac{N_j - N_i}{N_j + N_i}$$

$$t_{ij} = \frac{2N_i}{N_j + N_i}$$

The layer matrix (phase matrix) describing the propagation through layer j is described by

$$L_j = \begin{bmatrix} \exp(-i\beta_j) & 0 \\ 0 & \exp(i\beta_j) \end{bmatrix}$$

$$\beta_j = \frac{2\pi d_j N_j}{\lambda}$$

Where λ is the wavelength of the incident light and d is the layer thickness. All values are always calculated for each considered wavelength within layers. The reflectance and transmittance of the simulated sample from the front can be calculated using the ratio of input and output absolute electric fields

$$r = \frac{E_{0R}^{-}}{E_{0R}^{+}} = \frac{S_{21}}{S_{11}}$$

$$t = \frac{E_{(m+1)L}^{+}}{E_{0R}^{+}} = \frac{1}{S_{11}}$$

Similarly, the back side can be calculated accordingly

$$r' = \frac{E^{+}_{(m+1)L}}{E^{-}_{(m+1)L}} = -\frac{S_{12}}{S_{11}}$$

$$t' = \frac{E^{-}_{0R}}{E^{-}_{(m+1)L}} = \frac{\Delta S}{S_{11}}$$

$$\Delta S = S_{11}S_{22} - S_{12}S_{21}$$

The left boundary is calculated to consider the light in (E^{+}_{0R}) using the following relation

$$\begin{bmatrix} E^{+}_{jR} \\ E^{-}_{jR} \end{bmatrix} = S_j \begin{bmatrix} \frac{1}{S_{11}} \\ 0 \end{bmatrix} E^{+}_{0R}$$

And the right boundary similarly is defined as

$$\begin{bmatrix} E^{+}_{jR} \\ E^{-}_{jR} \end{bmatrix} = S_j \begin{bmatrix} -\frac{S_{12}}{S_{11}} \\ 1 \end{bmatrix} E^{-}_{(m+1)L}$$

In order to consider incoherent layers, the same approach can be taken to calculate scattering matrix of thicker layers but for its amplitudes

$$\begin{bmatrix} U^{+}_{0R} \\ U^{-}_{0R} \end{bmatrix} = \bar{S} \begin{bmatrix} U^{+}_{(m+1)L} \\ U^{-}_{(m+1)L} \end{bmatrix}.$$

Where $\bar{S}$ is calculated for incoherent layers

$$\bar{S} = \left(\prod_{v=1}^{m} \bar{I}_{(v-1)v} \bar{L}_v \right) \cdot \bar{I}_{m(m+1)}$$

And $\bar{I}$ is interface matrix between two incoherent layers

$$\bar{I}_{j(j+1)} = \frac{1}{\overline{|t|}} \begin{bmatrix} 1 & -|r'|^2 \\ \overline{|r|} & |tt'|^2 - |rr'|^2 \end{bmatrix}$$

Where power of two is used for transmittance and reflectance due to the consideration of amplitudes of electric field. Also there are defined as follows for front side coefficients

$$\bar{r} = \frac{U^{-}_{0R}}{U^{+}_{0R}} = \frac{\overline{S_{21}}}{\overline{S_{11}}},$$

$$\bar{t} = \frac{U^{+}_{(m+1)L}}{U^{+}_{0R}} = \frac{1}{\overline{S_{11}}},$$

$$\overline{r'} = \frac{U^{+}_{(m+1)L}}{U^{+}_{0R}} = -\frac{\overline{S_{12}}}{\overline{S_{11}}},$$

$$\bar{t}' = \frac{U^{-}_{0R}}{U^{-}_{(m+1)L}} = \frac{\overline{S_{11}S_{22}} - \overline{S_{12}\,S_{21}}}{\overline{S_{11}}}.$$

The propagation matrix $\bar{L}$ is defined

$$\bar{L_J} = \begin{bmatrix} \left|\exp(-i\beta_j)\right|^2 & 0 \\ 0 & \left|\exp(i\beta_j)\right|^2 \end{bmatrix},$$

where β_j defined the same as for coherent layers.

And link between light amplitude of electric field and electric field is

$$U = |E^2|.$$

Where U for each wavelength and layer is defined again as superposition

$$U_i(z) = U_i^-(z) + U_i^+(z)$$

The boundary conditions are defined in the same way as for coherent layers but for the parameters defined within incoherent layers.

Finally, to consider the mixture of coherent and incoherent layers within the simulated optical structure. We are taking the approach to first calculate all optical variables in coherent packet and use it for calculations of interface matrix between incoherent layers.

$$\overline{I_{\iota(\iota+1)}} = \begin{bmatrix} |S_{11}|^2 & -|S_{11}|^2 \\ |S_{21}|^2 & \dfrac{|\Delta S|^2 - |S_{12}S_{21}|^2}{|S_{11}|^2} \end{bmatrix}$$

$$\Delta S = S_{11}S_{22} - S_{12}S_{21}$$

Where all scattering matrixes are considered from coherent packet. Considering the total scattering matrix, we are able to calculate transmission, reflectance and absorption parameters in function of wavelength for each layer.

II Results

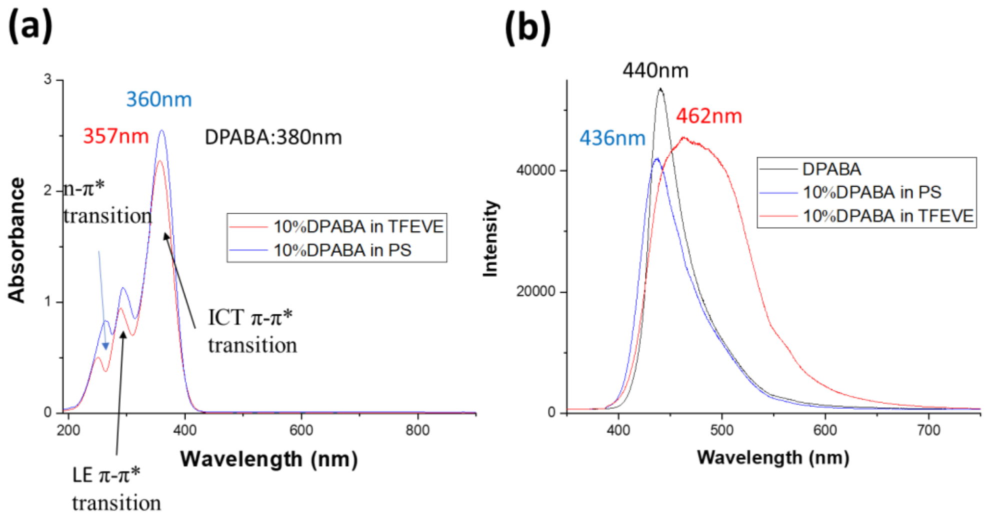

Figure S1 (A) Absorption, and (B) photoluminescence spectra of DPABA dispersing in PS and TFEVE respectively.

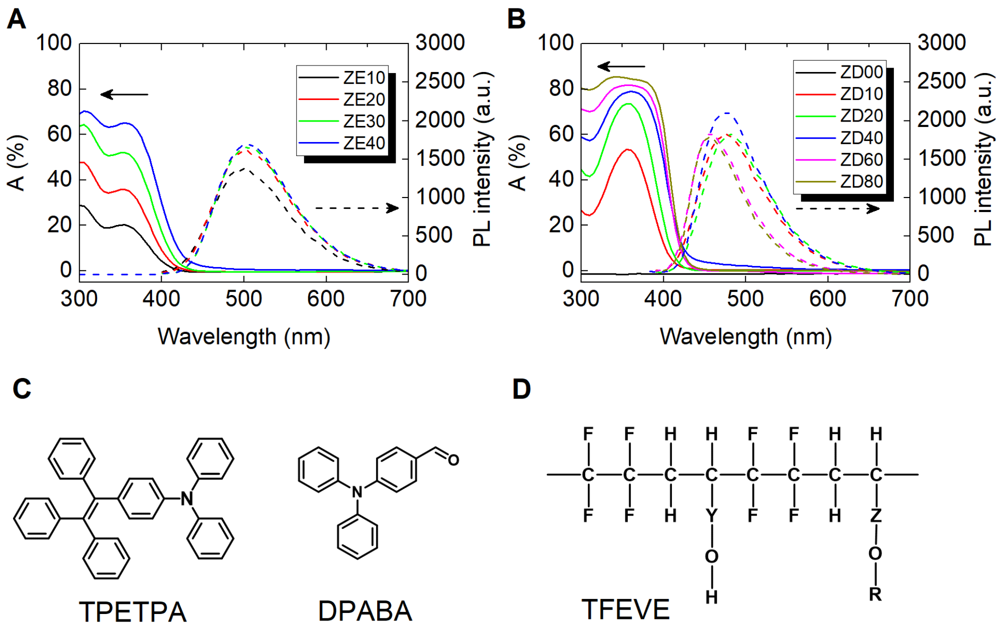

Figure S2 Absorption spectra and photoluminescent spectra for (A) ZE, and (B) ZD films containing different amount of down conversion molecule. The chemical structure of down-conversion molecules (C) TPETPA and DPABA molecules, and (D) binder TFEVE.

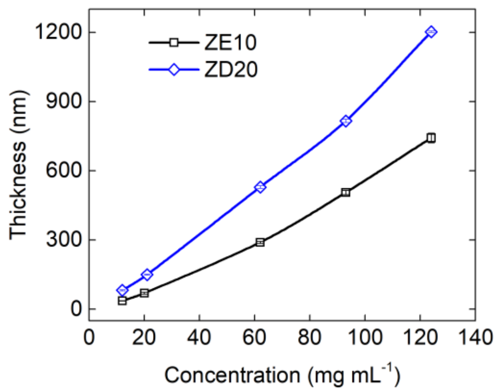

Figure S3 Thickness relation in ZE10 and ZD20 over the concentration of its solution respectively

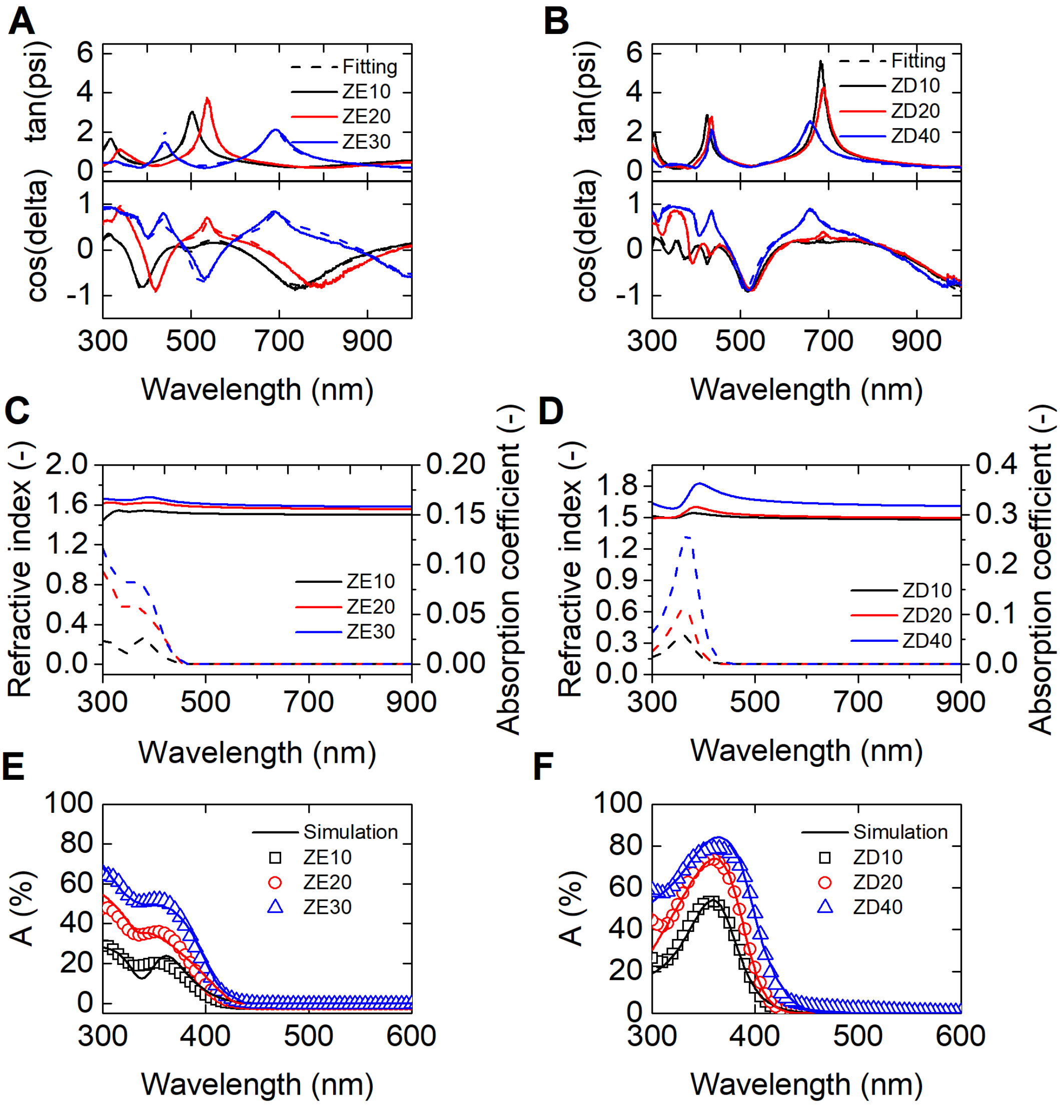

Figure S4 (A), (B) are ellipsometry and fitting results for ZE and ZD series. (B), (C) are n,k values from optical modeling for ZE and ZD series. (E), (F) are absorption spectra for experimental and optical simulation results for ZE and ZD series

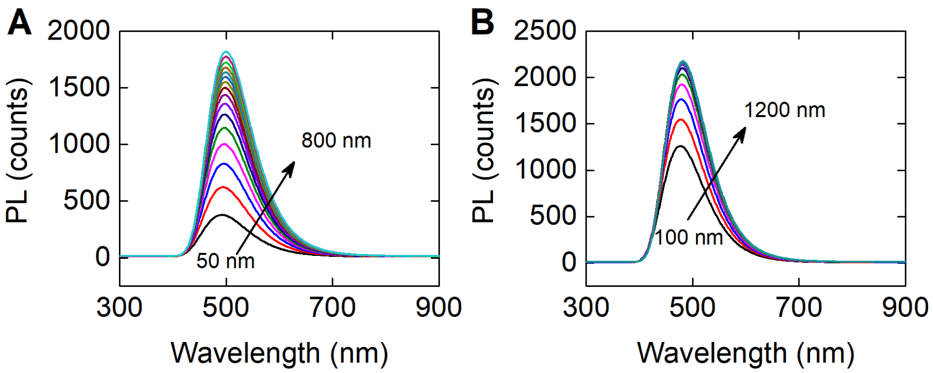

Figure S5 The PL thickness interpolation for the (A) ZE material in thickness from 50 nm to 800 nm, and (B) ZD material in thickness from 100 nm to 1200 nm

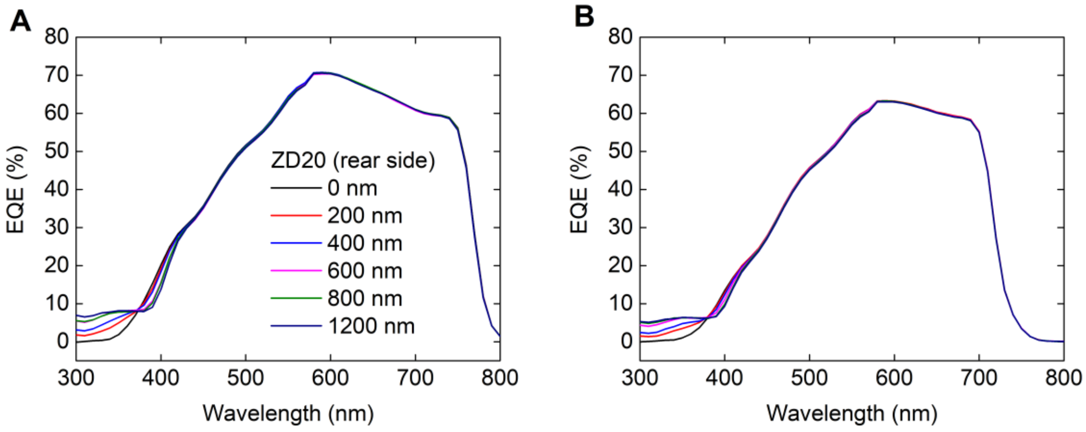

Figure S6 Plots of the changes of EQE of PSCs varied with the film thickness of different ZD20 (rear side) layer thicknesses on two (A and B) PSCs

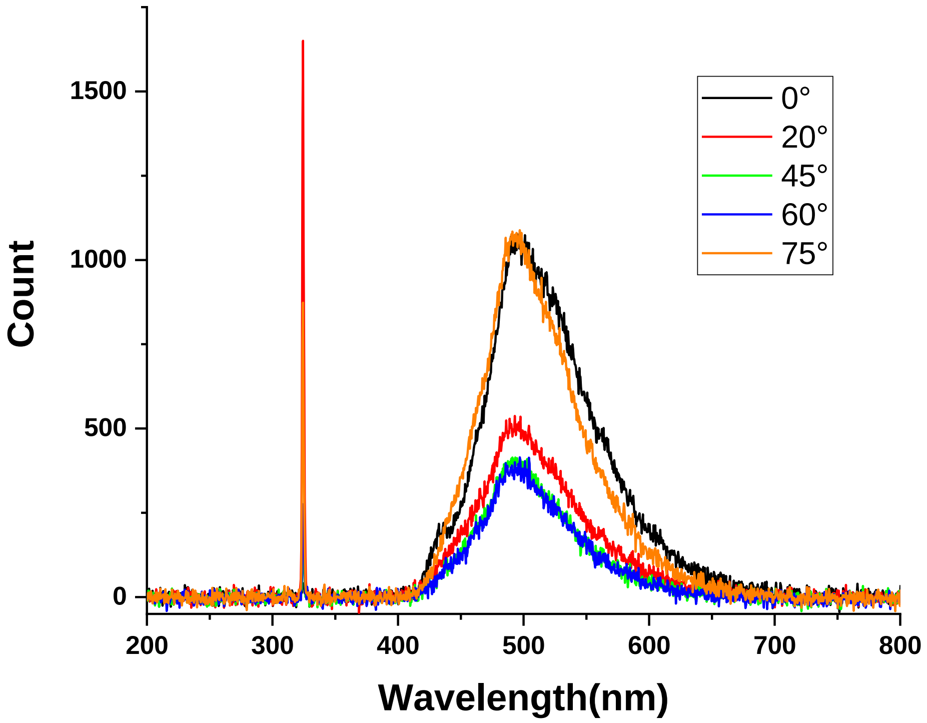

Figure S7 The PL measurements using variable angle detector for the ZD20 film illuminated with laser source with 325 nm wavelength.

Table S1 Compositions of ZE series materials and their corresponding thickness of the films with QY calculated based on PL emission

Sample	TFEVE in film (solid wt%)	TPETPA in film (solid wt%)	Total solid contention in THF* (mg/ml)	Film thickness † (nm)	PLQY (%)	Peak wavelength(nm)
ZE10	90	10	62	316.8	48	503

ZE20	80	20	62	319.0	48	503
ZE30	70	30	62	395.4	50	503
ZE40	60	40	62	-	51	504

*Total solid= wt% of TFEVE + wt% of TPETPA

† The thickness was measured by ellipsometer

Table S2 Different thickness of ZE10 films and their corresponding QY calculated based on PL emission

Sample	TFEVE in film (solid wt%)	TPETPA in film (solid wt%)	Total solid contention in THF* (mg/ml)	Film thickness† (nm)	PLQY (%)	Peak wavelength(nm)
ZE10-743	90	10	124	742.5±20.8	51	503
ZE10-506	90	10	93	506.0±9.6	49	503
ZE10/ ZE10-290	90	10	62	290.0±4.6	48	503
ZE10-70	90	10	20	69.8±4.8	44	499
ZE10-37	90	10	12	36.8±0.5	44	497

*Total solid= wt% of TFEVE + wt% of TPETPA

†The conc. of TPETPA is direct proportional to the thickness of the film.

Table S3 Composition of ZD series materials and their corresponding thickness of the films with QY calculated based on PL emission

Sample	TFEVE in film (solid wt%)	DPABA in film (solid wt%)	Total solid contention in EA* (mg/ml)	Film thickness † (nm)	PLQY (%)	Peak wavelength(nm)
ZD10	90	10	62	443.8	64	477
ZD20	80	20	62	439.2	60	480
ZD30	70	30	62	-	61	478
ZD40	60	40	62	373.2	64	476
ZD50	50	50	62	-	64	474
ZD60	40	60	62	396.7	51	457
ZD80	20	80	62	407.3	48	456

*Total solid= wt% of TFEVE + wt% of TPETPA

† The thickness was measured by ellipsometer

Table S4 Different thickness of ZD20 materials and their corresponding QY calculated based on PL emission

Sample	TFEVE in film (solid wt%)	DPABA in film (solid wt%)	Total solid contention in EA* (mg/ml)	Film thickness† (nm)	PLQY (%)	Peak wavelength(nm)

ZD20 – 1203	80	20	124	1202.6±2.9	61	482
ZD20 – 816	80	20	93	815.9±7.2	60	480
ZD20/ ZD20 – 529	80	20	62	529.3±5.6	60	480
ZD20 – 150	80	20	20	150.0±1.8	61	479
ZD20 – 82	80	20	12	82.1±2.4	60	479

*Total solid= wt% of TFEVE + wt% of TPETPA

†The conc. of DPABA is direct proportional to the thickness of film. The thickness was measured by profilometer.

Table S5. Vertical absorption (VA) and emission (VE) energies, oscillator strength (f), transition dipole moments (μ_{01}) and Stokes shifts calculated in a vacuum and in ethyl acetate with PCM (TDDFT/MN15-D3BJ/def2-TZVP).

Environment	VA (eV)	VA (nm)	f[a]	μ_{01} (a.u.)[a]	VE (eV)	VE (nm)	μ_{01} (a.u.)[b]	Stokes shift (eV)
DPABA								
Vacuum	3.73	332	0.471	2.27	2.93	422	0.90	-0.80
LR	3.59	345	0.623	2.66	2.99	414	2.55	-0.60
SS	3.42	362			2.42	512		-1.00
TPETPA								
Vacuum	3.42	362	0.526	2.51	1.92	646	2.51	-1.50
LR	3.38	366	0.627	2.75	1.68	736	3.21	-1.70
SS	3.29	377			1.80	689		-1.49

[a] Calculated at the S_0 geometry. [b] Calculated at the S_1 geometry.
